# Clinical Comparative Study of the Effects of Helicobacter Pylori Colonization on Oral Health in Children

**DOI:** 10.12669/pjms.324.10034

**Published:** 2016

**Authors:** Asim Dane, Taskin Gurbuz

**Affiliations:** 1 Asim Dane, Department of Pediatric Dentistry, Faculty of Dentistry, Izmir Katip Celebi University, Izmir, Turkey; 2 Taskin Gurbuz, Department of Pediatric Dentistry, Faculty of Dentistry, Ataturk University, Erzurum, Turkey

**Keywords:** Caries, CLO test, Gastritis, *Helicobacter pylori*, Pedodontics, Saliva

## Abstract

**Objective:**

To isolate Helicobacter pylori (HP) from the dental plaque of a selected group of children and to compare the oral and salivary findings of patients with those of a healthy control group.

**Methods:**

A total of 70 children aged 5–15 years were included in this study. An intraoral examination was performed for each patient, and dental plaque and saliva specimens were collected for analysis. Oral health conditions, nutritional habits, tooth brushing frequency, saliva pH levels, flow velocity, and buffering capacities were noted. The Kruskal–Wallis test was used for comparison of the DMFT and dft index. The significance level was set at p=0.05.

**Results:**

The prevalence of HP in dental plaque was higher in study group than controls (p<0.05). There were no significant differences between groups with respect to DMFT and dft scores, nutritional habits, tooth brushing frequency, saliva pH level or flow velocity (p>0.05). Meanwhile, the buffering capacity of saliva was lower in HP gastritis patients (p<0.05).

**Conclusion:**

There was a high prevalence of HP in dental plaque; thus, the oral cavity may be an important reservoir for HP. Good oral hygiene could be a positive contributor to the treatment of gastritis.

## INTRODUCTION

Many systemic diseases and oral manifestations are important for the early diagnosis of oral problems in children.^1^ Early symptoms of many systemic diseases occur in the oral cavity. Moreover, the oral cavity constitutes the first component of the gastrointestinal system.^2^ Thus, gastrointestinal diseases (GID) are crucial factors in determining oral health, since they may manifest in the oral cavity.

*Helicobacter pylori* (HP) is one of the most common causes of gastritis,^3^ which may be defined as an inflammation and erosion of the gastric mucosa. HP is a Gram-negative, rod-shaped, microaerophilic bacterium that is one of the major risk factors for gastritis, gastroduodenal ulcers, and gastric cancer.^4^ HP infection is quite common throughout the world, but a higher prevalence has been reported in developing countries.^5^ The prevalence of HP infection is extremely high in the Turkish population. In their study, Özden et al. reported HP-positive status in 79% of those aged 7–12, 83% of those aged 13–18, and 96% of those aged 25–29 years.^6^ In recent years, with the isolation of bacteria in dental plaque, the oral cavity has been promoted as a reservoir area, specifically, for example, the gastric mucosal membrane.^7^-^9^ Many studies have been performed on the presence of the HP in the gastric or non-gastric areas in adults, but there are only few such studies focusing on children. Furthermore, the results of HP detection in non-gastric areas are controversial.

The aim of this study was to determine the presence of HP in the dental plaque and to examine the relationship between the HP and dental caries (DMFT and dft scores), oral hygiene (plaque accumulation levels), nutritional habits, and salivary parameters (saliva pH level, flow velocity, and buffering capacity) in children.

## METHODS

The study group consisted of 35 patients who had endoscopically diagnosed cases of the HP gastritis and who were seen at the Department of Pediatric Gastroenterology, Hepatology and Nutrition, Ataturk University Medical School, Erzurum, Turkey. This study was approved by the Ethics Committee of the Ataturk University Institute of Health Sciences, Erzurum, Turkey (Ethics Committee Number: 2012.2.5). Thirty-five healthy patients comprised the control group. Patients who had been taking antibiotics and chlorhexidine preperations in the last four weeks before oral examination were excluded from the study. The patients ranged in age from 5 to 15 years. A standard protocol was employed that included variables like name, age, gender, sociodemographic status, nutritional and tooth brushing habits, oral examination, and salivary parameters.

### Sampling of dental plaque

We preferred to collect supragingival plaque samples because our patients were younger and their pocket depth was within the physiological limits. Dental plaque samples were obtained from the buccal, lingual, mesial, and distal parts of the maxillary first molar using sterile Gracey probes. The second molars or the second deciduous molars were sampled if the first molars were missing or had extensive restoration. Samples were collected after lunch but before cleaning of the oral cavity. The samples were immediately placed into the CLO test procedure (Kimberly-Clark, Roswell, USA) and the results were evaluated in one hour..

### Oral examination

The oral examination was carried out with a mouth mirror and probe under artificial light in the clinic using the World Health Organization (WHO) criteria (dft and DMFT indices).^10^ The oral examination included assessment of dental caries, oral hygiene status, plaque index, tooth brushing, nutritional habits and salivary pH, buffer capacity, and flow rate. Oral hygiene status was assessed and classified as good, fair, or poor using the Green and Vermillion’s oral hygiene index.^11^

The analysis of saliva was performed using Saliva-Check Buffer (GC Corp, Japan). Patients chewed paraffin wax for five minutes which was then collected in sterile cups. Following this, the saliva flow rate was recorded with the Saliva-Check Buffer with the following categorization: <3.5 ml was classed as very low, 3.5–5 ml as low, and >5 ml as normal. Similarly, buffer capacity was recorded using the same test. Sufficient saliva was dropped onto the test pads using a pipette. The buffer capacity was calculated according to the change in color of the test pads as follows: a score of 0–5 points was classified as very low, 6–9 points as low, and 10–12 points was classified as normal/high. The measurement of salivary pH was carried out with a pH test strip placed in the saliva sample for 10 s. The change in the color of the strip was compared with the testing chart in the package.

The dental plaque index was scored according to Silness and Loe’s classification, as follows:^12^ 0 = no plaque in the gingival area; 1 = a film of plaque adhering to the free gingival margin and adjacent area of the tooth; 2 = moderate accumulation of soft deposits within the gingival pocket, on the gingival margin, and/or adjacent to the tooth surface, which could be seen with the naked eye; and 3 = an abundance of soft matter within the gingival pocket and/or on the gingival margin and adjacent tooth surface. For the appropriate statistical analysis we considered a plaque score of 0.1–1.0 as mild, 1.1–2.0 as moderate, and 2.1–3.0 as severe. The tooth brushing habits and daily dietary habits (between meal snacking, etc.) were asked about to parents of the patients.

### Statistical analysis

The data were analyzed using SPSS for Windows (Release 18.0) statistical software. Kruskal–Wallis statistical analysis was used for comparison of the DMFT and dft index. The Chi-square test was used to analyze the data on HP in dental plaque and oral health status, where *p*-values of <0.05 were regarded as statistically significant.

## RESULTS

The study group consisted of 35 patients (18 females, 17 males), aged 5–15 years (mean: 9.71±2.49 years). The control group comprised 35 age- and sex-matched patients (mean age: 9.57±2.40 years). According to the CLO test procedure, 29 gastritis patients (82.9%) were dental plaque positive and 6 (17.1%) were negative; of the controls, 8 patients (22.9%) were positive, while 27 (77.1%) were negative. There was a significant difference between the groups. The incidence of HP in dental plaque was higher in gastritis patients than controls (*p*<0.05; Table I).

**Table-I:** The presence of oral HP in the groups.

Oral HP	Gastritis	Control
HP(+)	29	8
HP(-)	6	27

Significant difference between two groups, p<0.05.

The DMFT and dft scores of children with gastritis were determined as 4.37±1.41 and 1.97 ± 1.68, respectively. The control group’s DMFT and dft scores were 4.71±1.9 and 2.3±2.0, respectively. These results were statistically insignificant. The control group had a higher caries index result than the study group (*p*>0.05 for DMFT and dft). Six (17.1%) patients among the gastritis cases had good oral hygiene compared to 5 (14.3%) among controls. The oral hygiene status of 19 (54.3%) patients was classified as fair compared to 24 (68.6%) among controls, and 10 (28.6%) cases as poor compared to 6 (17.1%) among controls. The observed difference in the oral hygiene status between the two groups was not found to be statistically significant (*p*>0.05).

In the group of patient with gastritis, mild dental plaque accumulation was detected in 15 (42.9%), moderate in 17 (48.6%) and severe in 2 (5.7%) patients; one patient (2.9%) had no plaque. In the control patients, mild dental plaque accumulation was detected in 14 (40%), moderate in 12 (34.3%), and severe in 6 (17.1%) patients; three (8.6%) controls were found to have no plaque (Table II). Five patients (14.3%) in the study group had no brushing habits, and 20 (57.1%) reported irregular tooth brushing habits. Five patients (14.3%) brushed their teeth once a day and 5 (14.3%) twice a day. In the controls, 14 patients (40%) reported irregular tooth brushing; 10 of them (28.6%) brushed their teeth once a day, 7 (20%) twice a day, and 4 (11.4%) had no tooth brushing habits. The difference was not statistically significant between two groups (*p*>0.05; Table III).

**Table-II:** Plaque indices of patients groups.

PI	Gastritis		Control		p value

	n	%	N	%	
0	1	2.9	3	8.6	0,072
1	15	42.9	14	40	
2	17	48.6	12	34.3	
3	2	5.7	6	17.1	

**Table-III:** Frequency of brushing of patients groups.

	Gastritis		Control		p value

Frequency of brushing	n	%	n	%	
Once a day	5	14.3	10	28.6	
Twice a day	5	14.3	7	20	0.366
Irregular	20	57.1	14	40	
None	5	14.3	4	11.4	

In terms of the eating habits of gastritis patients, 23 (65.7%) consumed carbohydrates more than once or twice daily, while 26 (74.3%) did so in the control group. The difference was not statistically significant (*p*>0.05).

Of the pH levels in gastritis patients, only one (2.9%) patient had a “critical” level; the other 34 (97.1%) patients had “normal” pH levels. All controls had normal pH levels (*p*>0.05). In the study group, 13 children (37.1%) had normal stimulated salivary flow rates, whereas 20 children (54.3%) had normal rates in the control group (*p*=0.74). In the control group, 30 children (85.7%) had normal buffering capacities, and 17 gastritis patients (48.6%) had normal buffering capacities. The difference between the groups was statistically significant for buffering capacity (*p*<0.05; Table IV).

**Table IV:** Saliva parameters of two group.

	Gastritis		Control		

pH	n	%	n	%	p value
Critical	1	2.9	0	0	0.364
Normal	34	97.1	35	100	
*Saliva Flow Rate*					
Very Low	0	0	1	2.9	0.74
Low	22	62.9	13	37.1	
Normal	13	37.1	20	60	
*Saliva Buffer Capacity*					
Very Low	1	2.9	0	93.3	*0.04*
Low	17	48.6	5	14.3	
Normal-High	17	48.6	30	85.7	

## DISCUSSION

HP is known to be an agent of gastric infections, representing the highest infection prevalence in the world.^5^ It is colonized in different areas because of its chronic and persistent nature. The recurrence rate has increased, and even after eradication therapy with antibiotics, it has been reported that the bacteria causing the infection often recurs.^7^-^9^ The oral cavity and dental plaque may be a reservoir for HP infection.^13^ Reported positivity rates for HP in dental plaque in patients ranged from 60% to 90%.^14^ The results of the present study showed that HP was present in the dental plaque of 82.9% of the patients with gastritis. The present study’s findings, like those of other studies, demonstrated that HP is highly positive in dental plaque and may be a reservoir area for gastric re-infection.^7^, ^14^-^19^ In contrast with this study, it has been shown previously that dental plaque may not be a relevant reservoir of HP.^20^-^22^ This contradiction may be due to variations in patient populations, the complexity of the oral microbiota, and the condition of oral hygiene and HP infection.^23^

Song et al.^24^ reported that HP was present on molar teeth to a greater degree than on other teeth. This result can be explained according to HP’s microaerophilic characteristic. The exposure to oxygen was reduced in a stepwise manner from incisors towards the molars so that the HP could reproduce more frequently in the molar teeth.^25^ In our study, dental plaque samples were collected from the individuals’ molars.

In the literature, there have been many studies on the relationship between plaque index and the presence of oral HP.^8^,^13^,^26^-^32^ In many of these studies, the mean plaque index scores of study groups were not significantly different.^8^,^13^,^26^,^27^ The results of the present study are in agreement with these studies in that there was no significant difference in plaque index scores between the study group and controls. In contrast, some studies have reported plaque index scores of control groups that were significantly lower than those of the study groups.^29^,^31^ This difference could be due to variations in socioeconomic status, awareness of oral hygiene, and dietary habits.^8^

An association between oral *HP* infection and dental caries has been previously shown.^33^,^34^ In this study, we found no significant differences in terms of the caries index between the two groups. However, Liu et al.^29^ suggested that the HP in dental plaque may play a role in the occurrence of dental caries. Another study by Liu et al.^35^ showed that the HP infection in the oral cavity is associated with dental caries and poor dental hygiene. The possible reasons for these differences include different sampling methods, different sample populations, and distinctions in the infection status of the HP.

In the present study, oral hygiene habits were evaluated by assessing the frequency of tooth brushing and recorded dietary habits. It showed that the frequency of tooth brushing was not significantly different between the two groups. Similarly, Bali et al.^36^ found no significant difference in relation to the timing and frequency of tooth brushing. The absence of a significant difference does not indicate that patients’ oral hygiene status was good. Gastritis patients may be neglecting oral hygiene due to existing medical problems.

To the best of our knowledge, there have been no previous reports on the relationship between HP gastritis and salivary parameters. In the present study, there were no significant differences between groups in terms of salivary pH levels. The pH level of the saliva of patients with gastritis can be expected to be low because the pH indicator method is used for the measurement. If a pH meter is used, different results could be obtained. Namiot et al.^34^ reported that low salivary flow contributed to a reduction in the eradication of HP infection, especially in patients receiving drug therapy. The use of medications may contribute an inhibitory effect for the measurement of the salivary flow rate. In this study, there was no significant difference between the two groups in the salivary flow rate. It may be because none of the gastritis patients had started antibiotic treatment.

Statistically significant differences were found in terms of the buffering capacity of saliva between the patients with gastritis and controls. It was found to be lower in the gastritis patients. Medical problems can cause gastritis in patients with a low buffering capacity. As such further studies are required.

## CONCLUSION

High prevalence of HP in dental plaque has demonstrated that the oral cavity may be an important reservoir of the HP. Poor oral hygiene may cause reinfection after eradication therapy. Meanwhile, good oral hygiene could make a positive contribution to the treatment of gastritis. Thus, oral hygiene education should be given to patients. Further studies are necessary to clarify the significance of oral HP, as well as its relationship with salivary parameters and oral hygiene status.
